# Environment and gynaecologic cancers

**DOI:** 10.3389/or.2024.1430532

**Published:** 2024-10-08

**Authors:** Rudrika Chandra, Sarita Kumari

**Affiliations:** ^1^ Obstetrics and Gynaecology, Command Hospital, Panchkula, Haryana, India; ^2^ Department of Gynaecologic Oncology, National Cancer Institute, All India Institute of Medical Sciences, New Delhi, India

**Keywords:** gynaecologic cancer, environment, carcinogenesis, microbiota, infections

## Abstract

In the current era, environmental factors are well established as major causative agents for all cancers especially lung and breast cancer. We sought to review the current available literature on the topic pertaining to gynaecologic cancers. Although a few factors are well established in literature, others need more research to conclude.

## 1 Introduction

The global burden of gynaecologic cancers remains substantial, with significant disparities in incidence and outcomes based on geographic, economic, and social factors. Worldwide, there were 662,301 new cases of cervical cancer and 348,874 women died from the disease in 2022. It predominantly affects women in low- and middle-income countries due to limited access to screening and Human Papilloma Virus (HPV) vaccination with Sub-Saharan Africa, Latin America, and South Asia having the highest incidence rates. The incidence is projected to increase to 948,000 cases in 2050. Similarly, there were 324,603 new cases of ovarian cancer and 206,956 women died from the disease. Higher incidence is seen in developed countries such as North America and Europe, but mortality rates are high worldwide due to late-stage diagnosis. It is estimated that by 2050 the incidence will increase to 504,000 cases. For Uterine cancer, there were 420,368 new cases and 97,723 deaths and it is projected to increase to 676,000 new cases in 2050 (1). It is common in high-income countries and is attributed to higher prevalence of obesity and lifestyle factors. Vaginal and vulvar cancers are rare. Prevention through addressing the modifiable risk factors including environmental factors is essential to reduce the burden and improve women’s health worldwide.

All cancers develop as a result of mutation in certain genes. As per the Knudson’s Two Hit Hypothesis, two hits or mutations within a genome are necessary for a malignant phenotype to develop. In cancers with a hereditary basis, one allele is inherently mutated while the other allele acquires mutation during lifetime to develop into cancer. On the contrary, in sporadic cancers, both allelic mutations occur during the life span of an individual. Most solid tumours require 5–10 rate limiting mutations at various cancer susceptibility genes. The cancer susceptibility genes are either gate keeper genes, i.e., oncogenes and tumour suppressor genes or care taker genes, i.e., those involved in the DNA repair (2). Environmental factors influence and regulate carcinogenesis and this is widely supported by epidemiological and experimental studies. Up to 90% of cancers are due to environment and lifestyle factors. Only a small fraction of cancers (5%–10%) are attributed to germline mutations and even the penetrance of germline mutations may be modified by environmental factors (3). Prominent environmental factors implicated in carcinogenesis are socio-economic status, diet, physical activity, tobacco, alcohol, infections, radiation, medical drugs and hormones, chemical carcinogens (heavy metals) and air pollutants.

### 1.1 Environmental agents as carcinogens:

The International Agency for Research on Cancer (IARC) Monograph has classified the various environmental agents into four groups (Table 1) (4).

**TABLE 1:** Agents classified by the IARC Monograph Volumes 1–135.

Group 1	Carcinogenic to humans	128 agents
Group 2A	Probably carcinogenic to humans	95 agents
Group 2B	Possibly carcinogenic to humans	323 agents
Group 3	Not classifiable as to its carcinogenicity to humans	500 agents

Table 2 summarises the carcinogens by cancer site, i.e., related to female genital tract with sufficient or limited evidence.

**TABLE 2:** Carcinogens of female genital tract with sufficient or limited evidence.

Cancer site	Carcinogenic agents with sufficient evidence	Agents with limited evidence
Uterine cervix	HPV 16, 18, 31, 33, 35, 39, 45, 51, 52, 56, 58, 59 Tobacco smoking HIV 1 Diethylstilbestrol (exposure *in utero*) Estrogen–progestogen contraceptives	HPV 26, 53, 66, 67, 68, 70, 73, 82
Endometrium	Estrogen menopausal therapy Estrogen–progestogen menopausal therapy Tamoxifen	Diethylstilbestrol
Ovary	Asbestos (all forms) Estrogen menopausal therapy Tobacco smoking	Talc-based body powder (perineal use) X-radiation, gamma-radiation
Vulva	HPV 16	HIV 1 HPV 18, 33
Vagina	Diethylstilbestrol (exposure *in utero*) HPV 16	HIV 1

HPV, human papilloma virus; HIV, human immunodeficiency virus.

^a^ This table does not include factors not covered in the IARC Monographs, notably genetic traits, reproductive status, and some nutritional factors.

#### 1.1.1 Infections

##### 1.1.1.1 Human papilloma virus (HPV)

HPV is a non-enveloped, double stranded, circular Deoxyribonucleic Acid (DNA) virus with >100 types and of them, approximately 40 infect human genital tract (5). There are 15 oncogenic (high risk types) namely, 16, 18, 31, 33, 35, 39, 45, 51, 52, 56, 58, 59, 66, 68 and 70. Of them HPV 16 and 18 account for majority (70%) of cervical cancers (CC) worldwide and six other types (31, 33, 35, 45, 52, and 58) account for an additional 20%. Cervical HPV infection rates vary around the world, as does the number of infected women who go on to develop CC. HPV infection is also implicated as a causative factor in vaginal and vulvar cancers. Upto 90% of vaginal cancers and 50% of vulvar cancers are HPV associated (6). On an average, the worldwide prevalence of HPV in healthy women is 10% (range 6%–23%). More than 90% of infection is cleared in 2 years. A number of risk factors are known to increase the risks of HPV infection progressing to cancer including HIV coinfection, smoking as well as other genital tract infections. HPV is transmitted by both sexual and nonsexual routes and critical molecules for initiation and progression are oncoproteins E6 and E7 which act on p53 and pRB, respectively. E6 binds to p53 blocking apoptosis and E7 releases E2F from pRB driving cells into cycle (7).

##### 1.1.1.2 HPV, epigenetic modifications and cervico-vaginal microbiome

Epigenetic modifications, e.g., DNA methylation of L1, L2 and LCR genes of HPV leads to viral persistence and integration into epithelial cell; subsequently silencing of tumor suppressor genes, activation of oncogenes, and exacerbation of defects in DNA repair mechanisms. HPV induced non-coding RNAs are divided into three classes: (microsomal) miRNAs, long non-coding RNAs (lncRNAs), and circular non-coding RNAs (circRNAs). Aberrant expression of non-coding RNAs serves critical role in onset and progression of the disease. They affect signalling pathways like E6-p53, E7-pRb, PI3K-Akt, Notch and Wnt-β-catenin, amongst others (8). There are reports indicating the role of cervico-vaginal microbiome affecting the natural history of HPV infection. In the placebo arm of Costa Rica HPV vaccine trial, at visit 1, abundance of vaginal *Lactobacillus* was associated with clearance of incident hrHPV infections [Linear Discriminant Analysis (LDA) > 4.0], whereas Gardnerella was the dominant biomarker for hrHPV progression (LDA > 4.0). At visit 2, increased microbial diversity was significantly associated with progression to Cervical Intraepithelial Neoplasia (CIN)2+ (*p* = 0.027) (9). Emerging evidence shows that genital dysbiosis and/or specific bacteria might have an active role in the development and/or progression and metastasis of gynaecological malignancies, including CC through direct and indirect mechanisms, including modulation of oestrogen metabolism.

Cancer therapies might also alter microbiota at sites throughout the body. Reciprocally, microbiota composition can influence the efficacy and toxic effects of cancer therapies, as well as quality of life following cancer treatment. Modulation of the microbiome via probiotics or microbiota transplant might prove useful in prevention and improving responsiveness to cancer treatment and quality of life.

##### 1.1.1.3 Preventive aspect and role of HPV vaccination

HPV vaccine is a highly immunogenic with more than 98% recipients developing an antibody response to the virus after completion of vaccination course. Currently, three HPV vaccines: 9-valent HPV vaccine (Gardasil 9, 9vHPV), quadrivalent HPV vaccine (Gardasil, 4vHPV), and bivalent HPV vaccine (Cervarix, 2vHPV)—have been licensed by the U.S. Food and Drug Administration (FDA). All three HPV vaccines protect against HPV types 16 and 18 that cause most HPV cancers. To date, more than a billion doses of vaccine have been safely administered worldwide. Several nations have introduced HPV vaccination into their National immunization schedule since the approval of vaccine in 2006. Real world impact has been proven in population based observational studies. Falcaro et al. analysed the effects of the national HPV vaccination programme in UK, on CC and CIN3. The estimated relative reduction in CC rates by age at vaccine offer were 34% (95% CI 25–41) for age 16–18 years (school year 12–13), 62% (95% CI 52–71) for age 14–16 years (school year 10–11), and 87% (95% CI 72–94) for age 12–13 years (school year 8), compared with the reference unvaccinated cohort. The corresponding risk reductions for CIN3 were 39% (95% CI 36–41) for those offered at age 16–18 years, 75% (95% CI 72–77) for age 14–16 years, and 97% (95% CI 96–98) for age 12–13 years. The HPV immunisation programme in UK has successfully almost eliminated cervical cancer in women born since 1 September 1995 (10).

##### 1.1.1.4 Human Immunodeficiency Virus

Women living with HIV (WLHIV) are up to 7-times more likely to develop cervical/vaginal/vulvar cancer than uninfected women, the reasons being higher risk of co-infection with high-risk HPV types, HPV reactivation and persistence and low regression of HPV infection. CC is the most prevalent Acquired Immune Deficiency Syndrome defining malignancy in women. The prevalence of HPV infection is high in WLHIV, reported as 37.6%–41% which is much higher than the general population. The prevalence of high-grade neoplasia among HIV-positive women is higher, which is 6.4% as compared to 0.5% in HIV-negative women (11).

##### 1.1.1.5 Other sexually transmitted infections (STI)

Majority of Epithelial ovarian cancer (EOC) arises in fallopian tube whose epithelium undergoes damage and neoplastic transformation after pelvic inflammatory disease. In a nested case-control study within the European Prospective Investigation into Cancer and Nutrition (EPIC) cohort including 791 cases and 1,669 matched controls, past STI, particularly *Chlamydia trachomatis*, was associated with higher EOC risk. Positive serology to Pgp3 antibodies had a higher risk of mucinous histology (RR 2.30 [95% CI = 1.22–4.32]) whereas positive serology for heat shock protein 60 had a higher risk of serous histology (RR 1.44 [1.12–1.85]). Herpes Simplex Virus-2 was associated with higher risk of endometrioid histology (RR 2.35 [1.24–4.43]) (12).

#### 1.1.2 Hormones

##### 1.1.2.1 Combined hormonal contraceptives (CHC)

The association of hormonal contraceptive usage and CC is well known. In a review of 24 epidemiological studies which included 16,573 women with CC and 35,509 without disease; among current users of oral contraceptives, the risk of invasive CC increased with increasing duration of use (RR for 5 years of use versus never use, 1.90 [95% CI 1.69–2.13]). Risk declined after use ceased, and by 10 or more years had returned to that of never users (13).

On the contrary, usage of CHC (oral) confer a long term protection against ovarian cancer (OC) (*p* < 0.0001). In a meta-analysis including data from 45 epidemiological studies on 23,257 women with OC and 87,303 controls, the risk reduction was proportional to the duration of usage and persisted for more than 30 years after usage had ceased (14). Similarly CHC decreases the risk of endometrial cancer (EC) although to a lesser extent than OC. A systematic review of 15 case control and four large cohort studies demonstrated a decrease in the risk of endometrial cancer of about 50% for ever use of oral CHC and protective effect persisted for more than 10–20 years after cessation (15).

##### 1.1.2.2 Menopausal hormone therapy (MHT)

In the Million women study, 10 years usage of estrogen only MHT starting from the age of 50 conferred an excess risk of 10/1,000 cases of EC when compared to never users. Estrogen and tibolone increased the risk whereas progesterone counteracted the effects (16). Similarly women who use MHT are at an increased risk of both incident (RR 1.20 [95% CI 1.09–1.32; *p* = 0.0002]) and fatal OC (RR 1.23 [1.09–1.38; *p* = 0.0006]) with an increased risk for serous histology (17).

##### 1.1.2.3 Tamoxifen

The association between tamoxifen usage in breast cancer survivors and subsequent risk of endometrial cancer has been proven. In a comparative study, risk of endometrial cancer increased with longer duration of use; RR 2.0 (95% CI 1.2–3.2, *p* < 0.001) for 2–5 years and RR 6.9 (95% CI 2.4–19.4) for 5 years.

Stage III/IV was more common in long-term tamoxifen users (*p* = 0.006) and were more likely to have malignant mixed mesodermal tumours or sarcomas (*p* ≤ 0.02), p53+ (*p* = 0.05), and ER- (*p* ≤ 0.001) tumours.

3-year cancer-specific survival was worse for long term users (*p* = 0.02) (18).

##### 1.1.2.4 Diethylstilbestrol (DES)

DES is a synthetic estrogen which was prescribed to women between 1940 and 1971 to prevent miscarriage and premature labor. In late 90s reports indicated increased cancer risk in women exposed to DES *in utero*. In the largest cohort of 4,536 DES exposed and 1,544 unexposed daughters; three cases of vaginal clear cell adenocarcinoma occurred among the exposed daughters, resulting in a standardized incidence ratio of 40.7 (95% CI, 13.1–126.2) compared with population (19). This was sufficient to warrant a ban on the usage of this drug during pregnancy. Studies also indicated that *in utero* DES exposure increased the risk of high grade cervical intraepithelial neoplasia in upto 4% of exposed women.

#### 1.1.3 Cigarette smoke

A collaborative analysis of individual data on 13,541 women with CC and 23,017 women without cancer form 23 epidemiological studies suggested a RR of 1.69 (stratified by study and age at diagnosis) and 1.45 (CI 1.35–1.58) (stratified by study, age at diagnosis, number of sexual partners, duration of oral contraceptive usage, age at first intercourse and number of births) for squamous cell carcinoma. For adenocarcinoma of cervix the stratified RR was 0.9 (CI 0.78–1.07) (20). This suggests a causative role of smoking in CC particularly squamous cell carcinoma. Smoking influences the risk of CC in conjunction with HPV infection.

A recent meta-analysis of 109 studies provided a pooled RR of invasive cancer and preinvasive lesions respectively, of 1.70 (95% CI 1.53–1.88) and 2.11 (95% CI 1.85–2.39) for current versus never smokers, and, 1.13 (95% CI 1.02–1.24) and 1.29 (95% CI 1.15–1.46) respectively for former versus never smokers. The risk of CC increased with pack years and smoking duration and decreased with time since quitting (21).

In EOC, a similar risk is seen in published literature. Compared with never smokers, current smokers had significantly increased risk for mucinous tumors [HR = 1.85 (95% CI 1.08–3.16)] and those smoking more than 10 cigarettes per day had a doubling of risk [HR = 2.25 (95% CI 1.26–4.03)] (22).

On the contrary, the risk of EC seems to decrease with smoking. A meta-analysis suggested that ever smoking was significantly associated with a reduced risk of endometrial cancer among prospective studies (RR 0.81; 95% CI 0.74–0.88) and case control studies (OR 0.72; 95% CI 0.66–0.79) (23).

#### 1.1.4 Radiation exposure

Post radiation sarcoma of female genital tract is rare but a recognised event. Most reported cases have been associated with a history of radiotherapy for various gynaecologic cancers particularly after definite radiotherapy for CC. Although most cases are uterine sarcomas, angiosarcoma of lower genital tract is a rare endothelial malignancy associated with radiation exposure. Although cutaneous presentation is most common, they can arise in essentially any anatomic location, including superficial or deep soft tissue and visceral sites.

Angiosarcoma is a clinically aggressive entity, with a 5-year overall survival of 35% and a mean survival of approximately 7 months (24). In 2019, first case of cervical angiosarcoma post radiation exposure was reported. The patient had a history of radiotherapy for squamous cell carcinoma of the cervix 11 years prior (25).

There are few case reports of vaginal/vault angiosarcoma in the literature since 1980s. In a recent case report, a 54-year-old lady developed angiosarcoma of the vagina and vulva 9 years following radiotherapy for CC. She was treated with chemoradiotherapy and died 9 months following the diagnosis of angiosarcoma (26). Angiosarcomas should always be considered in the differential diagnosis when dealing with a tumour located in a previously irradiated area.

#### 1.1.5 Chemicals

Historically, an association was observed between asbestos use and risk of ovarian cancer. However careful interpretation of findings suggested that observed association is weak and inconsistent. Cases of peritoneal mesothelioma may have been misdiagnosed as ovarian cancer, and contributed to observed excesses (27).

There are few reports indicating that Bisphenol, a widely used raw material has estrogen like effects. It can stimulate proliferation of OVCAR-3 ovarian cancer cells after exposure for up to 5 days. This leads to an enhanced cell migration, invasion, and adhesion (28).

Genital talc use has been a debated causative agent for OC since long. In a pooled analysis of 8,525 cases and 9,859 controls, its use was associated with a modest increased risk of EOC (OR 1.24; 95% CI 1.15–1.33). Risk was elevated for invasive serous (OR 1.20; 95% CI 1.09–1.32), endometrioid (OR 1.22; 95% CI 1.04–1.43), clear cell (OR 1.24; 95% CI 1.01–1.52), and for borderline serous tumors (OR 1.46; 95% CI, 1.24–1.72) (29).

#### 1.1.6 Gut microbiome

Gut microbiome is constituted by the commensal micro-organisms which exist within the gastrointestinal tract and play an important role in health and disease. Next-generation sequencing and multi-“omics” technology has enhanced our understanding of the complex and interdependent relationship between the host and microbiome. Gut microbiome is influenced by multiple factors, i.e., diet, lifestyle, environment and they play an important role in carcinogenesis primarily through interactions with the immune system. However, there is a large gap in knowledge regarding its association with gynaecologic cancers.

Obesity, estrogen and inflammation have the potential to modify gut microbiome and impact gynaecologic cancers. A symbiotic gut flora has tumour suppressive effects by its anti-inflammatory, barrier function and antioxidant properties. Whereas a deviation in symbiosis, i.e., dysbiosis, leads to an oncogenic potential by creating inflammation, barrier failure and increased gut permeability, DNA damage, affecting estrogen metabolism and signalling pathways. In the estrogen-gut microbiome axis; estrogen metabolites in bloodstream enter enterohepatic circulation and undergo conjugation before entering the gastrointestinal system where estrogen metabolizing bacteria (estrobolome) deconjugate estrogen into active metabolites. Excess unconjugated estrogen undergoes reabsorption at peripheral sites resulting in DNA damage and tumorigenesis (30). Pre-clinical and clinical studies have demonstrate that specific microbial communities may be associated with increased risk for uterine, ovarian, and cervical cancers (31). Gut microbiome also affects immune checkpoint inhibitor efficacy in gynaecologic cancers and dietary interventions such as intermittent fasting/ketogenic diet, high fiber diet, use of probiotics could modulate the gut microbiome leading to changes in the tumour microenvironment (32). Conversely, cancer therapies might also alter microbiota at sites throughout the body eventually affecting the quality of life (33).

### 1.1.7 Diet, physical activity and caffeine

It is widely accepted that diet plays an important role in cancer development. However, the associations between dietary intake and gynaecological cancers remains unclear. A National Health and Nutrition Examination Survey (NHANES) was conducted from 2007 to 2016 in which 12,437 women aged over 20 years were included. The relationship between 30 dietary factors (4 macronutrients, 15 vitamins, 9 minerals, caffeine and alcohol) and gynaecological cancers were assessed. They observed negative correlation of intakes of phosphorus (OR: 0.998, 95% CI 0.996–0.999; *p* = 0.002) with CC, and intake of vitamin B12 (OR: 0.812, 95% CI 0.714–0.925; *p* = 0.002), phosphorus (OR: 0.997, 95% CI 0.996–0.999; *p* < 0.001) and alcohol (OR: 0.971, 95% CI 0.950–0.992; *p* = 0.009) with EC. The data showed positive association of intake of caffeine (OR: 1.002, 95% CI 1.001–1.003; *p* = 0.003) with CC, and intake of copper (OR: 2.754, 95% CI 1.313–5.778; *p* = 0.009) with EC. Intake of protein, total sugars, total fat, cholesterol, vitamin A, alpha-carotene, beta-carotene, beta-cryptoxanthin, lycopene, vitamin B2, niacin, vitamin B6, food folate, vitamin C, vitamin D, vitamin E, vitamin K, magnesium, iron and selenium showed no relationship with gynaecological cancers (*p* > 0.05). However, more epidemiological studies are needed to validate these results (34).

In the EPIC cohort 1,486 incident OC cases were identified. A positive association was found between OC and intake of industrial trans elaidic acid (HR 1.29; 95% CI 1.03–1.62; *p* = 0.02). Dietary intakes of n-6 linoleic acid (HR 1.10; 95% CI 1.01–1.21; *p* = 0.03) and n-3 α-linolenic acid (HR 1.18; 95% CI = 1.05–1.34; *p* = 0.007) from deep-frying fats were also positively associated. There is sufficient evidence to suggest a probable causal relationship with increased glycaemic load and EC and a probable inverse relation with coffee consumption (35).

Health benefits of physical activity has been demonstrated in gynaecologic cancers. In a meta-analysis there was a modest inverse association with levels of physical activity and OC. There is sufficient evidence to conclude that physical activity reduces the risk of EC (36).

#### 1.1.8 Air pollutants

The IARC has classified outdoor air pollution as a Group 1 carcinogen. Recent studies have reported an increased risk of gynaecologic cancer associated with air pollution. When exposed to air pollution, the respiratory tract is the primary damaged organ, but studies have confirmed that ultrafine particles can migrate through the blood to other organs and cause cancer. In a case–control study, authors examined the association of short-term exposure to air pollution with gynaecological cancer events using the clinical data of 35,989 women. The adjusted odds ratios (ORs) were examined to evaluate gynaecologic cancer risk in six time windows (Phase 1–Phase 6) of exposure to air pollutants (PM_2.5_, CO, O_3_, and SO_2_) and the highest ORs were found in Phase 4 (240 days). Higher adjusted ORs were found associated with increased concentrations of each pollutant (PM_2.5_, CO, O_3_, and SO_2_) in Phase 4. For instance, the adjusted OR for a 1.0-mg m^−3^ increase in CO exposures was 1.010 (95% CI: 0.881–1.139) below 0.8 mg m^−3^, 1.032 (95% CI: 0.871–1.194) at 0.8–1.0 mg m^−3^, 1.059 (95% CI: 0.973–1.145) at 1.0–1.4 mg m^−3^, and 1.120 (95% CI: 0.993–1.246) above 1.4 mg m^−3^. This study supports that the gynaecologic risks associated with air pollution should be considered in improved public health preventive measures and policymaking to minimize the dangerous effects of air pollution (37).

PM_2.5_ is also associated with a significant increase in risk of mortality for CC (RR 1.77, 95% CI 1.00–3.16) (38). Long-term exposure to air pollutants can induce oxidative stress reaction in cervical cells, consequently damaging DNA and presenting similar symptoms to HPV infection. This is similar to the effect of smoking. Air pollutant such as PM_2.5_ contain a variety of polycyclic aromatic hydrocarbons (PAHs) and its derivatives which is associated with genetic polymorphisms in activation of carcinogens and steroid hormone metabolism, thereby promoting the proliferation of cancer cells. Substantial studies are in favour of the linkage between the PAHs and CC. A recent study investigated the effects of PAHs exposure combined with hrHPV infection on CIN in community (N = 2,285). hrHPV infection (adjusted [aOR] = 4.08, 95% CI: 3.00–5.54), HPV16 infection (aOR = 4.71, 95% CI: 3.39–6.53), HPV58 infection (aOR = 2.29, 95% CI: 1.41–3.73) and PAHs high exposure (aOR = 2.57, 95% CI: 1.82–3.62) increased the risk of CIN2/3, showing an increasing trend (*p* < 0.001) with the severity of cervical lesions (39).

#### 1.1.9 Others

There is increasing evidence that chronic inflammation is involved in carcinogenesis and cancer progression. Recent data suggests that inhibition of inflammation through nonsteroidal anti-inflammatory drug (NSAID) use has therapeutic benefit for patients with colorectal cancer. Zheng et al. conducted two nationwide nested case-control studies among the Danish and Swedish female population, 11,874 women with OC (30–84 years old; Denmark: 7,328 diagnosed in 2000–2019, Sweden: 4,546 diagnosed in 2010–2018) were randomly age-matched with 473,960 female controls (293,120 from Denmark, and 181,840 from Sweden). They found that women with Ever use of low-dose aspirin was not strongly associated with the overall risk of OC (OR = 0.97; 95% CI: 0.92–1.03). However, the association differed according to parity (nulliparous: OR: 0.80, 95% CI 0.70–0.92; parous: OR: 1.00, 95% CI 0.94–1.07; *p* = 0.0024). The authors concluded that low-dose aspirin use was associated with a decreased OC risk especially in nulliparous women (40). Infertility has long been recognized as a risk factor for various cancers, including breast and gynaecologic cancers. More recently, concern has been raised regarding effects of drugs used to treat infertility, particularly since these drugs stimulate ovulation and raise endogenous estrogen levels. However, currently there does not appear to be an association between fertility drugs and cervical cancer. There is no conclusive evidence that fertility drugs increase the risk of uterine cancer, although women with infertility are at higher risk of uterine cancer. Women should be informed that there may be an increased risk of invasive and borderline ovarian cancers and thyroid cancer associated with fertility treatment. It is difficult to determine whether this risk is related to underlying endometriosis, or female infertility as such (41).

Hair products, i.e., dyes, straighteners, relaxers or pressing products, permanent or waves contain hazardous chemicals. A large cohort study with 10.9 years of follow up suggested that ever use of straightening products was associated with a higher incident uterine cancer rates (HR = 1.80, 95% CI = 1.12–2.88) (42).

Other than causation, environmental factors could be linked to an increase in mortality of diagnosed cases as well and a limited knowledge exists regarding the impact of multiple environmental factors on cancers in women. A study examining the association of Environmental quality index (five domains: air, water, land, built environment and sociodemographic domain) and mortality due to CC found that the only the sociodemographic index was negatively associated with CC mortality. The socioeconomic status (SES) largely determines the risk of developing lower genital tract cancer and plays a major role in survival too as it is linked to multiple other risk factors (43). Approximately 85% of women with CC live in a low middle income country (LMIC). Directly related to the SES is the educational background as it is linked to awareness regarding the disease and preventive measures.

Ongoing climate change may disrupt regular vaccination programs for CC but is unlikely to have a direct effect on HPV infection and other infectious causes (44).

## 2 Conclusion

**FIGURE 1:**
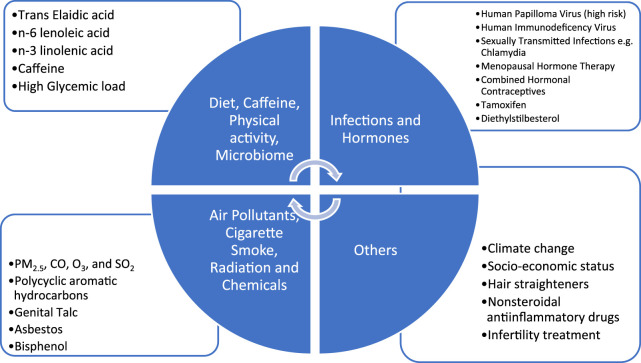
Environmental determinants of gynaecologic cancers.

The strongest association of lower genital tract cancers is with HPV infection. Primary prevention through vaccination and secondary prevention through screening can reduce incidence and mortality of these cancers. Strong association with hormonal exposure, endometrial and ovarian cancer is amenable to intervention. Decision for MHT should be individualised. There is an association of hormonal exposure (CHC) and cervical cancer although it appears to be an indirect one. Smoking cessation likely reduces the risk of cervical squamous cell carcinoma and mucinous ovarian cancer. Avoidance of talcum powder in the perineal region may have a modest effect on ovarian cancer risk. There is insufficient evidence to draw any strong conclusions regarding factors such as diet and physical activity, air pollutants however these are the areas for research. All the factors relevant for gynaecologic cancers have been depicted in Figure 1.
